# Low-Dose Aspirin Augments the Anti-Inflammatory Effects of Low-Dose Lithium in Lipopolysaccharide-Treated Rats

**DOI:** 10.3390/pharmaceutics14050901

**Published:** 2022-04-20

**Authors:** Rachel Shvartsur, Galila Agam, Sarit Uzzan, Abed N. Azab

**Affiliations:** 1 Department of Nursing, School for Community Health Professions, Faculty of Health Sciences, Ben-Gurion University of the Negev, P.O. Box 653, Beer-Sheva 8410501, Israel; shvartsu@post.bgu.ac.il; 2 Department of Clinical Biochemistry and Pharmacology, Faculty of Health Sciences, Ben-Gurion University of the Negev, P.O. Box 653, Beer-Sheva 8410501, Israel; galila@bgu.ac.il (G.A.); uzzan@post.bgu.ac.il (S.U.)

**Keywords:** aspirin, bipolar disorder, brain, inflammation, lithium, lipopolysaccharide

## Abstract

Mounting evidence suggests that immune-system dysfunction and inflammation play a role in the pathophysiology and treatment of mood-disorders in general and of bipolar disorder in particular. The current study examined the effects of chronic low-dose aspirin and low-dose lithium (Li) treatment on plasma and brain interleukin-6 and tumor necrosis factor-α production in lipopolysaccharide (LPS)-treated rats. Rats were fed regular or Li-containing food (0.1%) for six weeks. Low-dose aspirin (1 mg/kg) was administered alone or together with Li. On days 21 and 42 rats were injected with 1 mg/kg LPS or saline. Two h later body temperature was measured and rats were sacrificed. Blood samples, the frontal-cortex, hippocampus, and the hypothalamus were extracted. To assess the therapeutic potential of the combined treatment, rats were administered the same Li + aspirin protocol without LPS. We found that the chronic combined treatment attenuated LPS-induced hypothermia and significantly reduced plasma and brain cytokine level elevation, implicating the potential neuroinflammatory diminution purportedly present among the mentally ill. The combined treatment also significantly decreased immobility time and increased struggling time in the forced swim test, suggestive of an antidepressant-like effect. This preclinical evidence provides a potential approach for treating inflammation-related mental illness.

## 1. Introduction

In recent decades, mounting evidence has suggested that dysfunction of the immune system and inflammation, in general, and neuroinflammation, in particular, play a role in the pathophysiology of mental illnesses, including bipolar disorder (BD) [1,2,3,4,5,6]. Epidemiologic studies identified an elevated percentage of BD prevalence among patients with various inflammation-related comorbidities [7,8,9,10,11]. Nevertheless, the identified association has yet to be established as causal; the existing data implies that the association is bidirectional, presenting controversial elucidation over which phenomenon is the precursor [12]. A recent meta-analysis of 49 studies reported that BD patients, as compared to healthy control subjects, exhibited significantly elevated blood levels of C-reactive protein (CRP), interleukin (IL)-6 and tumor necrosis factor (TNF)-α [6]. When acute phases of the illness were considered separately, CRP and TNF-α were elevated in both depressive and manic episodes, but not in euthymia, while IL-6 remained elevated regardless of the disease state [6]. In cerebrospinal fluid (CSF) [13,14,15] and in the postmortem brain of bipolar patients [16,17], levels of inflammatory markers were found significantly higher as compared to control subjects. Many studies have shown that the therapeutic effects of psychotropic drugs, including mood stabilizers, antidepressants and antipsychotics exhibit, among others, anti-inflammatory properties [18,19,20,21,22]. Consistently, typical anti-inflammatory medications were observed as beneficial in clinical trials researching mood disorders [3,23,24,25,26,27,28,29].

Lithium (Li) is the traditional long-term maintenance therapy for BD [30]. It is useful in reducing the episodic incidence of mania and depression [30,31,32]. Additionally, Li lessens the rate of suicidal attempts and suicidal death in BD [33,34], particularly after long term use [35]. Two main hypotheses have been suggested to explain the therapeutic mechanism of action of Li: inositol monophosphatase-1 inhibition incurring consequential inositol depletion [36,37], and glycogen synthase kinase (GSK)-3β inhibition giving way to multiple cell signaling aftereffects [38,39]. However, despite appreciable recognition, neither theory has been accepted beyond doubt [40,41]. Li influences other cellular targets, affecting neurotransmission and second messenger signaling [42], enhancing autophagy [43,44], and impacting protein kinase C function [45], to name a few. In relevance to the present study, Li impacts countless immune and inflammatory processes [21,22,46,47], raising the likelihood that these effects may contribute to its pharmacological dynamics. For example, Li has been shown to mitigate prostaglandin (PG) levels in mammalian tissues including the brain via the inhibition of the arachidonic acid cascade [21,22]. The drug has also been reported to exert other anti-inflammatory effects such as the inhibition of cytokine production [48]. However, opposing findings, e.g., pro-inflammatory effects [22], have also been indicated. In this regard, discrepancies in experimental conditions, e.g., Li dose, model system, sex and strain of used animals, as well as other characteristics of the experimental model may account for the inconsistent results.

Aspirin is a nonsteroidal anti-inflammatory drug (NSAID) which exerts antiplatelet, analgesic, antipyretic and anti-inflammatory effects in a dose-dependent manner [49,50]. Aspirin (acetylsalicylic acid, ASA) inhibits the enzymes cyclooxygenase (COX)-1 and COX-2, leading to anti-inflammatory potence at high doses (>325 mg/day in adult humans) and antithrombotic/cardioprotective effects at lower doses (75–150 mg/day) [49,50]. The antithrombotic/antiplatelet feature of aspirin is attained through irreversible inhibition of COX-1, resulting in the reduction of platelet-derived thromboxane (TX) A2 production [49,50]. Beyond its recognized therapeutic efficacy in cardiovascular diseases [51,52,53], accumulating evidence suggests that low-dose aspirin confers anti-cancer properties [54,55,56,57]. Moreover, in the context of the present study, low-dose aspirin is known to affect immune cell function and inflammation [58]. Circumstantial evidence [59] premised the notion that the addition of low-dose aspirin to Li may increase the therapeutic effectiveness of Li. We recently reported [60] that co-administration of low-dose aspirin with low-dose Li in rats is safe and enhances the beneficial behavioral outcomes of low-dose Li without aggravating its toxicity. Add-on aspirin had a suppressive influence on Li-induced elevation in blood creatinine levels, indicative of a protective effect against Li nephrotoxicity. Taking into account the evidence attesting to the involvement of inflammation in the pathophysiology of mood disorders, and the results of our aforementioned study [60], we hypothesized that chronic treatment with low-dose aspirin plus low-dose Li would exhibit enhanced anti-inflammatory properties. Hence, the main objective of the present study was to examine the effects of the combination therapy on the production of inflammatory mediators in the rat model of lipopolysaccharide (LPS)-induced inflammation by measuring plasma and brain inflammatory mediators. In addition, to broaden the scope of mood-related behavioral effects of the combined treatment reported in our previous paper [60] (efficacy in the sucrose consumption test evaluating depressive-like behavior, and elevated plus maze and open field tests assessing anxiety-like behavior), we then studied the effects in the Porsolt’s forced-swim test (FST), modeling depressive-like behavior, and in the amphetamine-induced hyperactivity test (AHT), modeling manic-like behavior.

## 2. Materials and Methods

### 2.1. Animals

Male Sprague-Dawley rats weighing 220–250 g at the beginning of the experiments were used in the study. The inclusion of only male rats was thoroughly explained in the previous study [60] and derived from two main reasons: (1) Due to ethical considerations we strived to include the minimal possible number of animals in a “proof-of-concept” study; and, (2) the kinetics of Li varies between the genders [61], as well as the presentation and outcomes of animal models of disease [62,63,64] which would have complicated the statistical analysis of the results. Growth and maintenance conditions of the animals were fully described in the previous paper [60]. Briefly, animals were retained under regulated environmental conditions (ambient temperature 22 ± 1 °C, relative humidity 45–55%, photoperiod cycle 12 h light:12 h dark), with free access to food and water.

### 2.2. Chronic Treatment with Li and Aspirin

The treatment protocol was performed exactly as described in the previous study [60]. Rats were fed with regular or Li-containing food [0.1% (*w*/*w*)] for 42 days. This protocol produced Li plasma concentrations of 0.2–0.4 mEq/L [60]. Low-dose aspirin (1 mg/kg, intraperitoneally (ip) [60,65]), alone or together with Li, was administered for 42 days.

### 2.3. Inflammation Studies

#### 2.3.1. Induction of Inflammation

**Figure 1:**
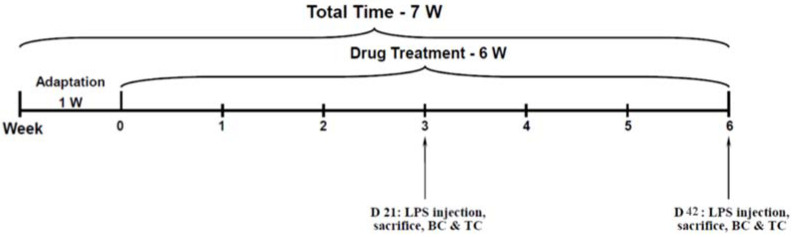
**Timeline of the LPS-induced inflammation experiment**. BC = blood collection; D = day; LPS = lipopolysaccharide; TC = tissue collection; W = week.

LPS (Sigma-Aldrich, St. Lewis, MO, USA) was suspended in a sterile saline 0.9% solution. On treatment days 21 and 42, at 2 h after the injection of aspirin, rats were injected ip with LPS 1 mg/kg to induce an inflammatory response of a mild magnitude [19]. A depiction of the timeline of the experiment is presented in Figure 1. At the same time, control rats were inoculated with sterile saline at equal volumes. In the inflammation studies, each experiment included eight groups (*n* = 16 rats per group): Control, Low-dose Li (Li), Aspirin (ASA), Li + ASA, LPS, LPS + Li, LPS + ASA, and, LPS + Li + ASA.

#### 2.3.2. Measurement of Body Temperature (BT)

BT was measured with a plastic-coated thermocouple probe (HL 600 Thermometer, Anristu Meter Co., Tokyo, Japan) inserted into the rectum. Rats were accustomed to this procedure before the initiation of the experimental protocol. Then, BT was measured every three days throughout the experiment to exclude the existence of pathological changes in BT and/or signs of inflammation before treatment with LPS. On the LPS induction day, BT was assessed before and at 2 h after LPS injection to detect LPS-induced changes in BT [19,48]. The LPS-induced changes in BT were calculated as the difference (delta) in BT between the value measured at 2 h after LPS injection to that measured before LPS injection, for each rat.

#### 2.3.3. Experimental Design

On treatment days 21 and 42, at 2 h after LPS injection, half of the animals in each group were euthanized by decapitation (see experimental timeline in Figure 1). Next, blood was collected in 15 mL tubes containing heparin and the frontal cortex (FC), hypothalamus (HT) and hippocampus (HC) were simultaneously and quickly excised and immediately frozen at −80 °C for later use [66,67]. Blood was centrifuged at 3500 rpm, 4 °C for 10 min, plasma was separated by aspiration using a tapered pipette inserted into the heparin-coated tubes, and kept at −80 °C until further use.

#### 2.3.4. Preparation of Brain Homogenates

Each brain region was weighed and manually homogenized on ice for 10 s in a buffer containing a protease/phosphatase inhibitor cocktail (1:10 *w*/*w*; Phosphatase Inhibitor Cocktail × 100 in ddH2O Catalog No. K1013, APExBIO; Protease Inhibitor Cocktail × 100 in DMSO, Catalog No. K1007, APExBIO). The homogenate was centrifuged at 10,000 rpm, 4 °C for 10 min, and then supernatants and pellets were separated and immediately frozen at −80 °C [66,67].

#### 2.3.5. Evaluation of IL-6 and TNF-α Levels

IL-6 and TNF-α levels were examined using specific enzyme-linked immunosorbent assay (ELISA) kits (R&D Systems, Minneapolis, MN, USA; Catalog numbers DY506 and DY510, respectively). The lowest detection limit of the IL-6 and TNF-α assays were 125 and 62.5 pg/mL, respectively. For samples in which the level of the examined constituent was under the lowest detection limit of the assay, results were categorized as “undetectable” and calculated as zero. Importantly, all brain samples were homogenized in the same amount of a homogenizing buffer (550 µL) irrespective of their weight. Therefore, levels of IL-6 and TNF-α were calculated as follows: ELISA result in pg/mL divided by the sample weight in milligrams. In the relevant figures, results are presented as: pg/mg wet weight.

### 2.4. Behavioral Studies

The behavioral studies were performed using the same Li and aspirin protocol, but without the induction of inflammation by LPS (to avoid LPS-induced behavioral changes). Thus, in the behavioral studies, each experiment included four groups (*n* = 12 rats per group): Control, Low-dose Li (Li), Aspirin (ASA), and, Li + ASA. All behavioral experiments were performed throughout the light phase, and were initiated at 2 h after the administration of aspirin.

#### 2.4.1. Experimental Design

**Figure 2:**
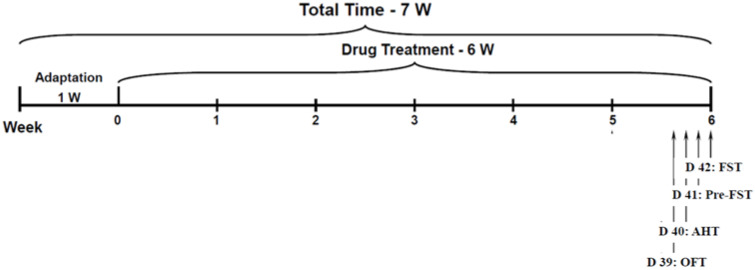
**Timeline of the behavior experiments.** AHT = amphetamine-induced hyperactivity test; D = day; FST = forced swim test; OFT = open field test; W = week.

Rats were treated with Li and aspirin as described in Section 2.2. On day 39 of the treatment protocol, rats were assessed for spontaneous locomotor activity in the open field test. On day 40, the AHT was performed to assess hyperactive/mania-like behavior. On days 41 and 42, rats were subjected to FST sessions. In the behavioral experiments, all treatment groups included 12 rats per group. A depiction of the timeline of the behavioral experiments is presented in Figure 2.

#### 2.4.2. Open Field Test (OFT)

The OFT was utilized to evaluate the spontaneous locomotor activity of animals [68]. Rats were positioned in the corner of an open field made of a black box (60 cm [W] × 80 cm [L] × 60 cm [H]) and were left there for 20 min. Sessions were videotaped by a camera positioned approximately 1 m above the center of the arena and afterwards evaluated using a video-tracking system (Ethovision, Noldus, Wageningen, The Netherlands). Only the last 10 min of the sessions were analyzed; the initial 10 min were regarded as adaptation time. The apparatus was cleaned before the introduction of each animal using a 5% ethanol solution. The parameters evaluated were total distance traveled and mean velocity of movement.

#### 2.4.3. Amphetamine-Induced Hyperactivity Test (AHT)

This is a widely utilized model for assessing hyperactive/mania-like behavior in rodents [68,69,70]. On day 40 of the treatment protocol (at the minimum of 2 h after the administration of aspirin), rats were injected ip with amphetamine (0.5 mg/kg) and were allowed to stay in their home cage for 30 min. Immediately thereafter, they were placed in the corner of an open field for 20 min to measure locomotor activity exactly as described in Section 2.4.2. Only the last 10 min of the sessions were analyzed; the initial 10 min were regarded as adaptation time. The open field arena was cleaned as described above (Section 2.4.2). Total distance traveled and mean velocity were analyzed by the video-tracking system.

#### 2.4.4. Porsolt’s Forced Swim Test (FST)

The FST is an accepted model for the assessment of depressive-like behavior in rodents [71,72,73]. The test examines immobility/floating time and swimming/struggling time. Immobility time represents despair and passive-like behavior is identified as the time when the rat floats in the water and makes only the necessary movements to keep its head above the water. Swimming/struggling time represents active/non-depressive behavior. On day 41 of the treatment protocol, a 2 min pretest FST session was conducted. Rats were placed to swim inside a glass cylinder (height 100 cm, diameter 40 cm) filled halfway with water at room temperature (24–28 °C). On day 42, rats were subjected to a 5 min test session, during which their behavior was filmed and later analyzed by the video-tracking system. The duration of immobility and struggling was analyzed during the last 4.5 min of the 5 min test; the initial 30 s were regarded as adaptation time.

### 2.5. Statistical Analyses

**Figure 3:**
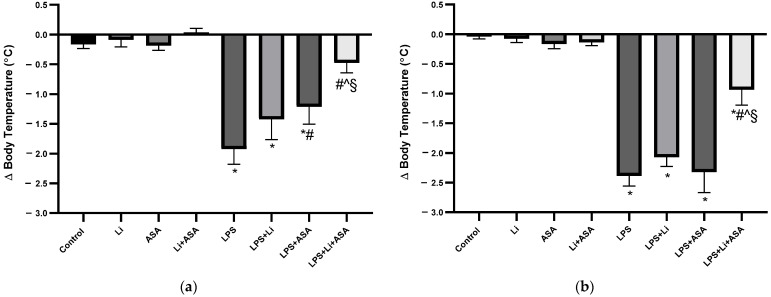
**Effects of Li and aspirin co-administration on LPS-induced hypothermia.** Rats were fed regular food (control) or Li-containing food (0.1%) for 42 days. Low-dose aspirin (1 mg/kg, ip) was given alone or together with Li. On days 21 (**a**) and 42 (**b**) of the treatment protocol, at 2 h before sacrifice, LPS (1 mg/kg) was administered as described under Materials and Methods. BT was measured before and 2 h post LPS injection. The bars show the difference (delta) in BT between the two measurements. Results are means ± SEM of a representative experiment out of two demonstrating a similar pattern, with eight rats/group. (**a**) Day 21: One-way ANOVA, F = 13.24, *p* < 0.0001. Post-hoc Fisher’s LSD test: Control vs. LPS, LPS + Li and LPS + ASA, *p* < 0.006, Control vs. LPS + Li + ASA, NS; LPS vs. LPS + Li, *p* = 0.08; LPS vs. LPS + ASA and LPS + Li + ASA, *p* < 0.016; LPS + Li vs. LPS + ASA, NS; LPS + Li vs. LPS + Li + ASA, *p* = 0.0016; LPS + ASA vs. LPS + Li + ASA, *p* = 0.013. (**b**) Day 42: One-way ANOVA, F = 36.43, *p* < 0.0001. Post-hoc Fisher’s LSD test: Control vs. LPS, LPS + Li, LPS + ASA and LPS + Li + ASA, *p* < 0.0007; LPS vs. LPS + Li and LPS + ASA, NS; LPS vs. LPS + Li + ASA, *p* < 0.0001; LPS + Li vs. LPS + ASA, NS; LPS + Li vs. LPS + Li + ASA, *p* < 0.0001; LPS + ASA vs. LPS + Li + ASA, *p* < 0.0001. Asterisks and symbols denote the following: * *p* < 0.05 vs. Control; # *p* < 0.05 vs. LPS; ^ *p* < 0.05 vs. LPS + Li, § *p* < 0.05 vs. LPS + ASA. Abbreviations: ASA—acetylsalicylic acid, Li—lithium, LPS—lipopolysaccharide, NS—nonsignificant.

**Figure 4:**
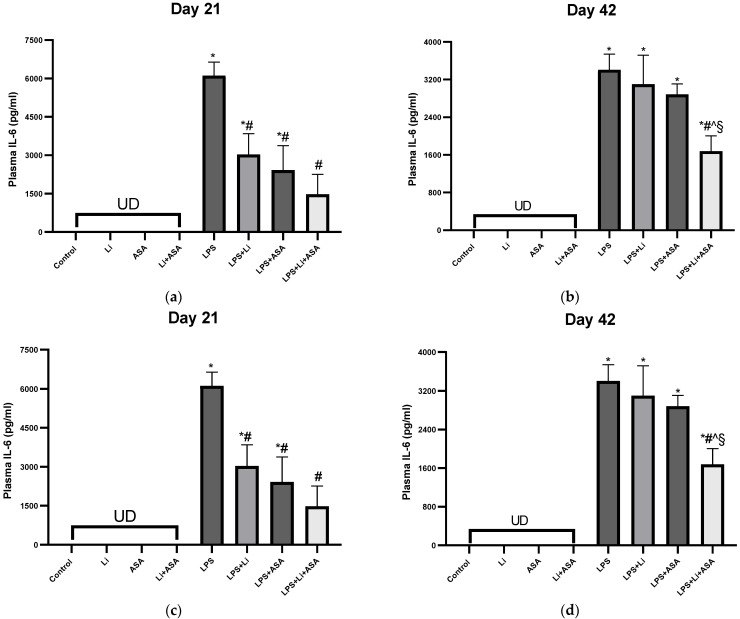
**Effects of Li and aspirin co-administration on plasma levels of IL-6 and TNF-α.** Rats were fed regular food (control) or Li-containing food (0.1%) for 42 days. Low-dose aspirin (1 mg/kg, ip) was given alone or together with Li. On days 21 (**a**,**c**) and 42 (**b**,**d**) of the treatment protocol, 2 h before sacrifice, LPS (1 mg/kg) was administered as described under Materials and Methods. Determination of IL-6 and TNF-α was done using specific ELISA kits. Results are means ± SEM of a representative experiment out of two demonstrating a similar pattern, with eight rats/group. (**a**) Plasma IL-6 on day 21: One-way ANOVA, F = 15.53, *p* < 0.0001. Post-hoc Fisher’s LSD test: Control vs. LPS, LPS + Li and LPS + ASA, *p* < 0.003; Control vs. LPS + Li + ASA, NS; LPS vs. LPS + Li, LPS + ASA and LPS + Li + ASA, *p* <0.0002; LPS + Li vs. LPS + ASA, NS; LPS + Li vs. LPS + Li + ASA, *p* = 0.052; LPS + ASA vs. LPS + Li + ASA, NS. (**b**) Plasma IL-6 on day 42: One-way ANOVA, F = 33.90, *p* < 0.0001. Post-hoc Fisher’s LSD test: Control vs. LPS, LPS + Li, LPS + ASA and LPS + Li + ASA, *p* < 0.0001; LPS vs. LPS + Li and LPS + ASA, NS; LPS vs. LPS + Li + ASA, *p* < 0.0001; LPS + Li vs. LPS + ASA, NS; LPS + Li vs. LPS + Li + ASA, *p* = 0.005, LPS + ASA vs. LPS + Li + ASA, *p* = 0.0018. (**c**) Plasma TNF-α levels on day 21: One-way ANOVA, F = 14.23, *p* < 0.0001. Post-hoc Fisher’s LSD test: Control vs. LPS, LPS + Li, LPS + ASA and LPS + Li + ASA, *p* < 0.03; LPS vs. LPS + Li and LPS + ASA, NS; LPS vs. LPS + Li + ASA, *p* = 0.0006; LPS + Li vs. LPS + ASA, NS; LPS + Li vs. LPS + Li + ASA, *p* = 0.001; LPS + ASA vs. LPS + Li + ASA, *p* = 0.021. (**d**) Plasma TNF-α levels on day 42: One-way ANOVA, F = 15.55, *p* < 0.0001. Post-hoc Fisher’s LSD test: Control vs. LPS, LPS + Li, LPS + ASA and LPS + Li + ASA, *p* < 0.03; LPS vs. LPS + Li, LPS + ASA, NS; LPS vs. LPS + Li + ASA, *p* < 0.0001; LPS + Li vs. LPS + ASA, NS; LPS + Li vs. LPS + Li + ASA, *p* = 0.007; LPS + ASA vs. LPS + Li + ASA, *p* = 0.007. Asterisks and symbols denote the following: * *p* < 0.05 vs. Control, # *p* < 0.05 vs. LPS; ^ *p* < 0.05 vs. LPS + Li; § *p* < 0.05 vs. LPS + ASA. Abbreviations: ASA—acetyl salicylic acid; Li—lithium, LPS—lipopolysaccharide, NS—nonsignificant, UD—undetectable.

**Figure 5:**
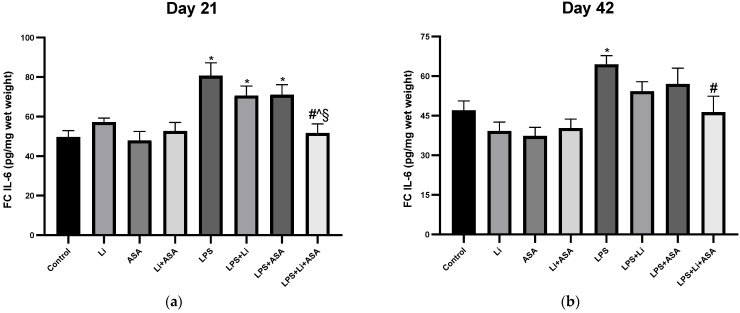

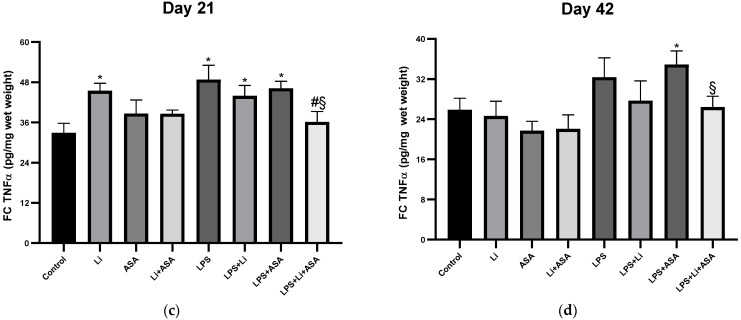
**Effects of Li and aspirin co-administration on frontal cortex levels of IL-6 and TNF-α.** Rats were fed regular food (control) or Li-containing food (0.1%) for 42 days. Low-dose aspirin (1 mg/kg, ip) was given alone or together with Li. On days 21 (**a**,**c**) and 42 (**b**,**d**) of the treatment protocol, 2 h before sacrifice, LPS (1 mg/kg) was administered as described under Materials and Methods. Determination of IL-6 and TNF-α was done using specific ELISA kits. Results are means ± SEM of a representative experiment out of two demonstrating a similar pattern, with eight rats/group. (**a**) FC IL-6 levels on day 21: One-way ANOVA, F = 7.199, *p* < 0.0001. Post-hoc Fisher’s LSD test: Control vs. LPS, LPS + Li and LPS + ASA, *p* < 0.002; Control vs. LPS + Li + ASA, NS; LPS vs. LPS + Li and LPS + ASA, NS; LPS vs. LPS + Li + ASA, *p* < 0.0001; LPS + Li vs. LPS + ASA, NS; LPS + Li vs. LPS + Li + ASA, *p* = 0.005; LPS + ASA vs. LPS + Li + ASA, *p* = 0.004. (**b**) FC IL-6 levels on day 42: One-way ANOVA, F = 5.131, *p* < 0.0001. Post-hoc Fisher’s LSD test: Control vs. LPS, *p* = 0.005; Control vs. LPS + Li, LPS + ASA and LPS + Li + ASA, NS; LPS vs. LPS + Li and LPS + ASA, NS; LPS vs. LPS + Li + ASA, *p* = 0.0037; LPS + Li vs. LPS + ASA and LPS + Li + ASA NS; LPS + ASA vs. LPS + Li + ASA, NS. (**c**) FC TNF-α levels on day 21: One-way ANOVA, F = 3.323, *p* = 0.005. Post-hoc Fisher’s LSD test: Control vs. Li, LPS, LPS + Li and LPS + ASA, *p* < 0.013; Control vs. LPS + Li + ASA, NS; LPS vs. LPS + Li, LPS + ASA, NS; LPS vs. LPS + Li + ASA, *p* = 0.0047; LPS + Li vs. LPS + ASA and LPS + Li + ASA, NS; LPS + ASA vs. LPS + Li + ASA, *p* = 0.023. (**d**) FC TNF-α on day 42: One-way ANOVA, F = 2.559, *p* = 0.023. Post-hoc Fisher’s LSD test: Control vs. LPS, LPS + Li, NS; Control vs. LPS + ASA, *p* = 0.032; Control vs. LPS + Li + ASA, NS; LPS vs. LPS + Li, *p* = 0.262; LPS vs. LPS + ASA, LPS + Li + ASA, NS; LPS + Li vs. LPS + ASA and LPS + Li + ASA, NS; LPS + ASA vs. LPS + Li + ASA, *p* = 0.043. Asterisks and symbols denote the following: * *p* < 0.05 vs. Control, # *p* < 0.05 vs. LPS; ^ *p* < 0.05 vs. LPS + Li; § *p* < 0.05 vs. LPS + ASA Abbreviations: ASA—acetylsalicylic acid, Li—lithium, LPS—lipopolysaccharide, NS—nonsignificant.

**Figure 6:**
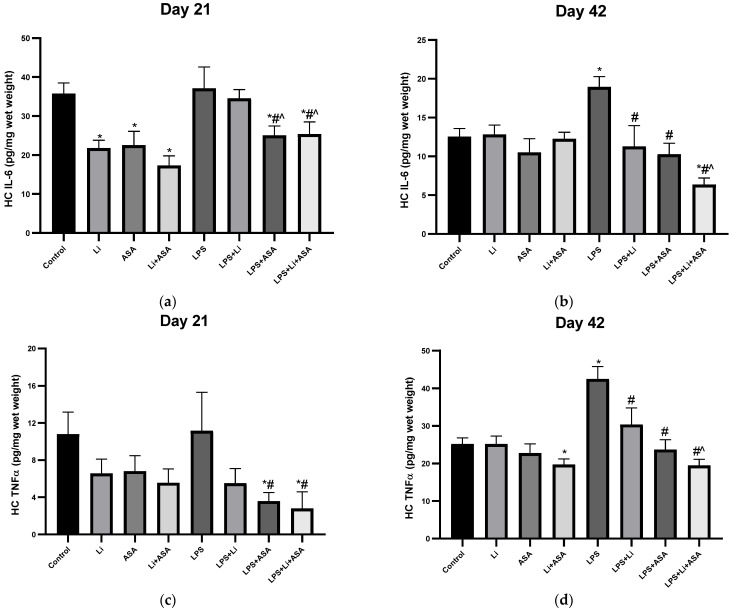
**Effects of Li and aspirin co-administration onhippocampal levels of IL-6 and TNF-α.** Rats were fed regular food (control) or Li-containing food (0.1%) for 42 days. Low-dose aspirin (1 mg/kg, ip) was given alone or together with Li. On days 21 (**a**,**c**) and 42 (**b**,**d**) of the treatment protocol, 2 h before sacrifice, LPS (1 mg/kg) was administered as described under Materials and Methods. Determination of IL-6 and TNF-α was done using specific ELISA kits. Results are means ± SEM of a representative experiment out of two demonstrating a similar pattern, with eight rats/group. (**a**) HC IL-6 levels on day 21: One-way ANOVA, F = 5.421, *p* < 0.0001. Post-hoc Fisher’s LSD test: Control vs. Li, ASA and Li + ASA, *p* < 0.003; Control vs. LPS and LPS + Li, NS; Control vs. LPS + ASA and LPS + Li + ASA, *p* < 0.024; LPS vs. LPS + Li, NS; LPS vs. LPS + ASA and LPS + Li + ASA, *p* < 0.011; LPS + Li vs. LPS + ASA and LPS + Li + ASA, *p* < 0.045; LPS + ASA vs. LPS + Li + ASA, NS. (**b**) HC IL-6 levels on day 42: One-way ANOVA, F = 5.439, *p* < 0.0001. Post-hoc Fisher’s LSD test: Control vs. LPS and LPS + Li + ASA, *p* < 0.005; Control vs. LPS + Li and LPS + ASA, NS; LPS vs. LPS + Li, LPS + ASA and LPS + Li + ASA *p* < 0.0002; LPS + Li vs. LPS + ASA, NS; LPS + Li vs. LPS + Li + ASA, *p* = 0.025; LPS + ASA vs. LPS + Li + ASA, NS. (**c**) HC TNF-α levels on day 21: One-way ANOVA, F = 2.270, *p* = 0.042. Post-hoc Fisher’s LSD test: Control vs. Li, Li + ASA, LPS, LPS + Li, NS; Control vs. LPS + ASA and LPS + Li + ASA, *p* < 0.01; LPS vs. LPS + Li, NS; LPS vs. LPS + ASA and LPS + Li + ASA, *p* < 0.013; LPS + Li vs. LPS + ASA and LPS + Li + ASA, NS; LPS + ASA vs. LPS + Li + ASA, NS. (**d**) HC TNF-α on day 42: One-way ANOVA, F = 8.160, *p* < 0.0001. Post-hoc Fisher’s LSD test: Control vs. LPS, *p* < 0.0001; Control vs. LPS + Li, LPS + ASA and LPS + Li + ASA, NS; LPS vs. LPS + Li, LPS + ASA and LPS + Li + ASA, *p* < 0.002; LPS + Li vs. LPS + ASA, NS; LPS + Li vs. LPS + Li + ASA, *p* = 0.005; LPS + ASA vs. LPS + Li + ASA, NS. Asterisks and symbols denote the following: * *p* < 0.05 vs. Control, # *p* < 0.05 vs. LPS; ^ *p* < 0.05 vs. LPS + Li. Abbreviations: ASA—acetylsalicylic acid, Li—lithium, LPS—lipopolysaccharide, NS—nonsignificant.

**Figure 7:**
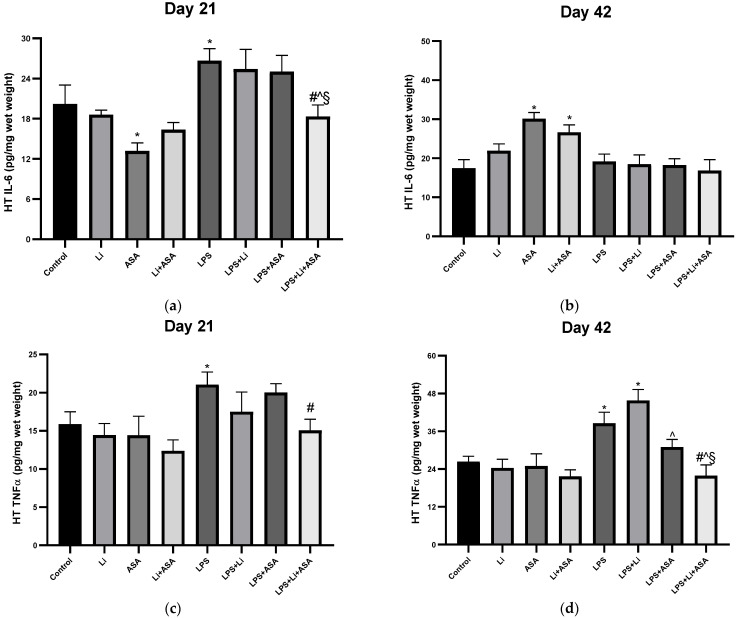
**Effects of Li and aspirin co-administration on hypothalamic levels of IL-6 and TNF-α**. Rats were fed regular food (control) or Li-containing food (0.1%) for 42 days. Low-dose aspirin (1 mg/kg, ip) was given alone or together with Li. On days 21 (**a**,**c**) and 42 (**b**,**d**) of the treatment protocol, 2 h before sacrifice, LPS (1 mg/kg) was administered as described under Materials and Methods. Determination of IL-6 and TNF-α was done using specific ELISA kits. Results are means ± SEM of a single representative experiment out of two demonstrating a similar pattern, with eight rats/group. (**a**) HT IL-6 levels on day 21: One-way ANOVA, F = 5.847, *p* < 0.0001. Post-hoc Fisher’s LSD test: Control vs. Li, Li + ASA, LPS + Li, LPS + ASA and LPS + Li + ASA, NS; Control vs. ASA, *p* = 0.015; Control vs. LPS, *p* = 0.025; LPS vs. LPS + Li, LPS + ASA, NS; LPS vs. LPS + Li + ASA, *p* = 0.004; LPS + Li vs. LPS + ASA, NS; LPS + Li vs. LPS + Li + ASA, *p* = 0.015; LPS + ASA vs. LPS + Li + ASA, *p* = 0.02. (**b**) HT IL-6 levels on day 42: One-way ANOVA, F = 5.566, *p* < 0.0001. Post-hoc Fisher’s LSD test: Control vs. ASA and Li + ASA, *p* < 0.0025; Control vs. Li, LPS, LPS + Li, LPS + ASA and LPS + Li + ASA, NS; LPS vs. LPS + Li, LPS + ASA, LPS + Li + ASA, NS; LPS + Li vs. LPS + ASA and LPS + Li + ASA, NS; LPS + ASA vs. LPS + Li + ASA, NS. (**c**) HT TNF-α levels on day 21: One-way ANOVA, F = 2, 714, *p* = 0.017. Post-hoc Fisher’s LSD test: Control vs. Li + ASA, LPS + Li, LPS + ASA and LPS + Li + ASA, NS; Control vs. LPS, *p* = 0.047; LPS vs. LPS + Li and LPS + ASA, NS; LPS vs. LPS + Li + ASA, *p* = 0.022; LPS + Li vs. LPS + ASA and LPS + Li + ASA, NS; LPS + ASA vs. LPS + Li + ASA, NS. (**d**) HT TNF-α on day 42: One-way ANOVA, F = 8.325, *p* < 0.0001. Post-hoc Fisher’s LSD test: Control vs. Li + ASA, LPS + ASA and LPS + Li + ASA, NS; Control vs. LPS and LPS + Li, *p* < 0.0058; LPS vs. LPS + Li, LPS + ASA, NS; LPS vs. LPS + Li + ASA, *p* = 0.0002, LPS + Li vs. LPS + ASA and LPS + Li + ASA, *p* ≤ 0.001; LPS + ASA vs. LPS + Li + ASA, *p* = 0.037. Asterisks and symbols denote the following: * *p* < 0.05 vs. Control, # *p* < 0.05 vs. LPS; ^ *p* < 0.05 vs. LPS + Li, § *p* < 0.05 vs. LPS + ASA. Abbreviations: ASA—acetylsalicylic acid, Li—lithium, LPS—lipopolysaccharide, NS—nonsignificant.

**Figure 8:**
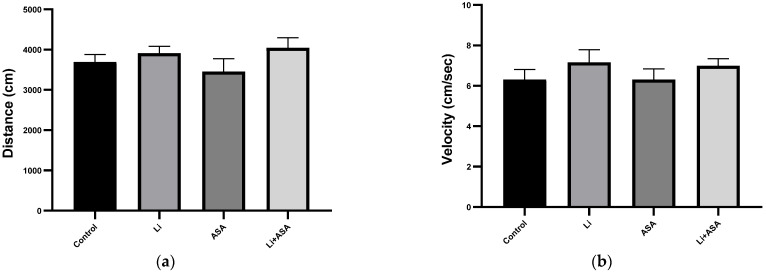
**Effects of Li and aspirin co-administration on amphetamine-induced hyperactivity.** On day 40 of the treatment protocol, rats were injected with amphetamine (as described in Materials and Methods) after which they were placed in an open field arena for 20 min. Total distance traveled (**a**) and mean velocity (**b**) during the last 10 min of the sessions were assessed by a video-tracking system. Results are means ± SEM of a single representative experiment out of two demonstrating a similar pattern, with 12 rats per group. (**a**) Total distance traveled: one-way ANOVA, *p* = NS. (**b**) Mean velocity: one-way ANOVA, *p* = NS. Abbreviations: ASA—acetylsalicylic acid, Li—lithium, NS—nonsignificant.

**Figure 9:**
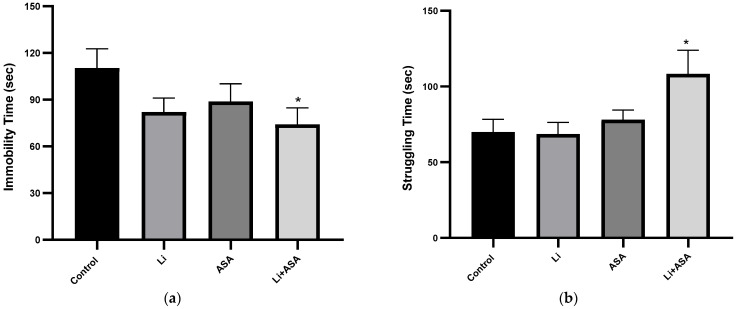
**Effects of Li and aspirin co-administration on rats’ performance in the FST.** On day 42 of the treatment protocol, rats were subjected to a 5 min FST session. Rats’ behavior was videotaped and subsequently analyzed by a video-tracking system. Immobility time and struggling time were measured as described in Materials and Methods. Results are means ± SEM of a single representative experiment out of two demonstrating a similar pattern, with 12 rats per group. (**a**) Immobility time: one-way ANOVA, *p* = NS. Student’s-*t* test for the difference between the Control and the Li + ASA group, *p* = 0.0235. (**b**) Struggling time: one-way ANOVA, *p* = 0.029. Post-hoc Fisher’s LSD test: Control and the Li + ASA group, F = 3.298, *p* = 0.011. Asterisks and symbols denote the following: * *p* < 0.05 vs. Control. Abbreviations: ASA—acetylsalicylic acid, FST—forced swim test, Li—lithium, NS—nonsignificant.

Firstly, normality was confirmed and, accordingly, appropriate statistical tests were used to determine statistical significance. Namely, differences between the groups were analyzed by one-way analysis of variance (ANOVA) or Student’s *t*-test, and the Fisher’s post-hoc test was used for between-group comparisons. Values of *p* < 0.05 were considered statistically significant. All results are expressed as means ± SEM. For each parameter presented, two independent experiments were performed. As mentioned, in the inflammation studies, the initial number of rats in each group was 16, which were divided into two sub-groups (*n* = 8 per group), one sacrificed on day 21 and one on day 42 (Figure 1). Thus, in the inflammation-related figures (Figure 3, Figure 4, Figure 5, Figure 6 and Figure 7), each group represents eight rats. In the behavioral studies, each group represents 12 rats (Figure 8 and Figure 9). Therefore, the total number of animals used in the entire study was 352 rats, taking into account that each experiment was performed twice.

## 3. Results

### 3.1. Inflammation Experiments

#### 3.1.1. Effect of Li and Aspirin Co-Administration on LPS-Induced Hypothermia

Treatment with LPS alone led to a significant decrease in BT (hypothermia) at 2 h post injection both on day 21 and day 42 of the experimental protocol (Figure 3). Low-dose Li (alone), low-dose aspirin (alone) and their combination did not alter BT in control (non LPS-treated) rats. In LPS-treated rats, chronic pretreatment with low-dose Li did not affect LPS-induced hypothermia, while low-dose aspirin significantly reduced the hypothermia on day 21 of the treatment. Importantly, add-on aspirin to Li significantly attenuated LPS-induced hypothermia both on day 21 and day 42 of the treatment protocol, suggestive of an enhanced thermo-modulating effect of the medications (Figure 3). As seen, the magnitude of LPS-induced hypothermia was significantly lower in aspirin plus Li-treated rats as compared to Li-only or aspirin-only treated rats (Figure 3).

#### 3.1.2. Effect of Li and Aspirin Co-Administration on Plasma Levels of IL-6 and TNF-α

No inflammatory cytokines were detected in the blood of control non LPS-treated rats (Figure 4). Treatment with LPS led to a significant increase in plasma IL-6 and TNF-α levels 2 h post injection both on day 21 and day 42 of the treatment protocol (Figure 4). Treatment with Li or aspirin (each by themselves) for 21 days significantly attenuated the LPS-induced increase in plasma IL-6 (Figure 4a). Treatment with Li + aspirin led to a more prominent decrease in IL-6 levels as compared to the monotherapies, however, the difference between the groups was not significant (Figure 4a). On day 42, only the combination treatment was associated with a significant reduction in IL-6 levels in LPS-treated rats (Figure 4b), indicative of an augmented anti-inflammatory effect of this treatment regimen. As to TNF-α, once again, on day 21, only the combination treatment was associated with a significant reduction in TNF-α levels in LPS-treated rats (Figure 4c). Similarly, on day 42, only the combination treatment caused a significant decrease in plasma TNF-α levels in LPS-treated rats (Figure 4d), supporting our hypothesis that the combination treatment produces an enhanced anti-inflammatory effect.

#### 3.1.3. Effect of Li and Aspirin Co-Administration on Frontal Cortex IL-6 and TNF-α Levels

LPS treatment significantly increased FC IL-6 levels on days 21 and 42 of the treatment protocol (Figure 5a,b) and only the combination treatment significantly counteracted the effect of LPS, attesting to an enhanced anti-inflammatory effect of the combination treatment. Of note, on day 21, the combination treatment reduced FC IL-6 levels to a significantly lower quantity than that of the monotherapy groups (Figure 5a). On the other hand, an inconsistent trend was obtained with the various treatments on FC TNF-α levels (Figure 5c,d). On day 21, LPS (by itself or with Li or ASA as monotherapy) significantly increased FC TNF-α levels. Unexpectedly, Li by itself also led to a similar direction of change. The combination treatment significantly counteracted the effect of LPS (Figure 5c). On day 42, LPS treatment by itself resulted in a trend of increased FC TNF-α levels which did not reach statistical significance and was intensified by the addition of aspirin (Figure 5d). No differences among the groups were observed in non LPS-treated rats (Figure 5a–d) except, as mentioned above, a significant Li-induced increase in TNF-α levels in the 21-day experiment.

#### 3.1.4. Effect of Li and Aspirin Co-Administration on IL-6 and TNF-α Hippocampal Levels

On day 21 of the treatment protocol, treatment with Li or aspirin, each by itself, or their combination resulted in a significant reduction in HC IL-6 levels in control non LPS-treated rats (Figure 6a). LPS did not significantly alter IL-6 levels. Aspirin significantly decreased HC IL-6 levels in LPS-treated rats (Figure 6a). A similar trend was obtained for HC TNF-α levels on day 21 (Figure 6c). Namely, Li, aspirin and their combination caused a non-significant reduction in TNF-α levels in non LPS-treated rats (Figure 6c). On day 42, LPS treatment significantly increased HC IL-6 and TNF-α levels (Figure 6b,d, respectively), which was diminished by independent administration of Li or aspirin as monotherapy (Figure 6b). For both cytokines, the combination treatment caused a more prominent effect (reduction) than the monotherapies in LPS-treated rats (Figure 6b,d, respectively), reaching statistical significance as compared with the effect of Li. Overall, these results also attest to an enhanced anti-inflammatory effect of the combined treatment. In non LPS-treated rats, no differences among the groups were observed for IL-6 levels in the 42-day experiment (Figure 6b), and for TNF-α levels in both experiments (21 and 42 days, Figure 6c,d), except for a significant Li + aspirin-induced decrease in TNF-α levels in the 42-day experiment.

#### 3.1.5. Effect of Li and Aspirin Co-Administration on Hypothalamic Levels of TNF-α and IL-6

On day 21 of the treatment protocol, administration of aspirin by itself significantly reduced HT IL-6 levels in non LPS-treated rats (Figure 7a). LPS significantly increased both IL-6 levels and TNF-α, an effect which was counteracted by the combined treatment with Li + aspirin (Figure 7a,c). Surprisingly, on day 42, both aspirin by itself and combined Li + aspirin significantly increased HT IL-6 levels in control rats (Figure 7b). LPS significantly elevated HT TNF-α levels, which was counteracted by the combination treatment (Figure 7d). Overall, in the HT, only the combination treatment managed to downregulate LPS-induced elevation of the two inflammatory markers.

### 3.2. Behavioral Experiments

As mentioned above, inflammation has been extensively linked to the pathophysiology and treatment of various psychiatric illnesses, particularly mood disorders [1,2,3,4,5,6,74]. In our previous study [60] we showed that co-treatment with low-dose Li and low-dose aspirin was associated with beneficial behavioral effects, including an anxiolytic-like effect (as modeled in the elevated plus maze test and OFT) and an antidepressant-like effect (as modeled in the sucrose consumption test). In the present study and following the observations attesting to enhanced anti-inflammatory effects of the combination therapy (Figure 3, Figure 4, Figure 5, Figure 6 and Figure 7), we strived to expand the scope of the previous behavioral analysis [60] and further elucidate the behavioral effects of the combination treatment, focusing on mood-disorder-modeling tests. To this end, we used the AHT for modeling hyperactive/mania-like behavior (Figure 8) and the FST for modeling depressive-like behavior (Figure 9).

#### 3.2.1. Effects of Li and Aspirin Co-Administration on Amphetamine-Induced Hyperactivity

On day 39 (the day before conducting the AHT), rats were subjected to a 20 min OFT session to assess their spontaneous activity and detect/exclude alterations in normal locomotor activity [68]. There were no significant differences between the groups in total distance traveled or mean velocity of movement (data not shown). On day 40 (the day of the AHT), administration of amphetamine significantly increased the distance traveled and mean velocity in control animals as compared to the previous day (distance: 3692 ± 166 vs. 2179 ± 190, respectively, *p* < 0.001; mean velocity: 6.31 ± 0.49 vs. 4.21 ± 0.38, respectively, *p* < 0.005). Chronic low-dose Li treatment either by itself or in combination with low dose aspirin did not affect amphetamine-induced hyperactivity (Figure 8a,b).

#### 3.2.2. Effect of Li and Aspirin Co-Administration on Rats’ Performance in the FST

On day 42 of the treatment protocol, rats were subjected to an FST. As seen in Figure 9a, chronic treatment with low-dose Li or low-dose aspirin, each by themselves, reduced the immobility time of the rats, but this trend did not reach statistical significance. As for the struggling time, neither of the drugs, when administered individually, affected outcomes (Figure 9b). On the other hand, add-on aspirin to Li significantly decreased the immobility time (Figure 9a) and increased the struggling time (Figure 9b), interpretable as an antidepressant-like effect.

## 4. Discussion

The present study tested the possibility that low-dose Li and low-dose aspirin exert augmented anti-inflammatory and beneficial mood-related behavioral effects in rats. The major findings of the study are: (1) co-treatment with low-dose Li and low-dose aspirin attenuated the severity of LPS-induced hypothermia and diminished the elevation in plasma and brain cytokine levels, and, (2) the combination treatment decreased immobility time and increased struggling time in the FST, indicative of an antidepressant-like effect. These results suggest that the combination therapy led to significantly more prominent anti-inflammatory and anti-depressant-like effects than each medication as a monotherapy (Li or aspirin).

In rodents, treatment with bacterial endotoxins leads to neuroinflammation and pathological BT changes [19,46,48,75,76,77]. Namely, systemic LPS administration causes a brief (~2 h) hypothermic period followed by a rise in BT (fever) [48,76,78]. Consistent with previous studies [19,48,76,78,79], we observed significant hypothermia following LPS injection, which was notably mitigated by aspirin monotherapy after 21 (but not 42) days, but not by Li monotherapy. As we had hypothesized, the combination therapy markedly attenuated the hypothermia both on days 21 and 42 of the treatment protocol (Figure 3). Using different treatment protocols, we have previously reported attenuation of LPS-induced hypothermia by Li [19,48]. In the present study, Li was administered orally and chronically in a regimen designed to produce plasma Li levels of 0.2–0.4 mEq/L [60], while in the previous studies, Li (100 mg/kg) was administered ip either acutely [48] or chronically [19] to produce plasma concentrations regarded as therapeutically-relevant in clinical practice. For example, in the study of Nassar et al. [19], the treatment regimen resulted in plasma Li concentration of 0.69 ± 0.16 mEq/L [Nassar A & Azab AN; personal communication], within the accepted therapeutic range in bipolar patients (0.6–1.2 mEq/L) [80,81]. It is conceivable that the relatively low plasma concentrations of the drug in the present study are responsible for the failure to mitigate the LPS-induced hypothermia, and that higher concentrations are required to achieve anti-hypothermic protection.

It is not understood how LPS leads to a biphasic febrile response in rodents. It is thought that the induced BT fluctuations result from increased synthesis of inflammatory constituents, such as PGE2, TNF-α, IL-1β and leukotrienes [19,48,76,79,82]. In this context, several studies suggested that COX-1 is pertinent in the development of the initial LPS-induced hypothermia, while the succeeding fever is attributed to COX-2 [76,78,83,84]. Thus, Steiner et al. [83] reported that a selective COX-1 inhibitor prevented LPS-induced hypothermia, while a selective COX-2 inhibitor enhanced the hypothermia but prevented the fever [83]. This corroborates the protective effect of low-dose aspirin against LPS-induced hypothermia in our study (Figure 3a), given that low-dose aspirin mainly inhibits COX-1 [49,50].

Multiple studies found an association between inflammation and brain function. Marsland et al. [85] showed that peripheral inflammation was linked to a cognitive decline by way of impacting brain morphology. An inverse association between IL-6 levels as well as total gray matter and hippocampal volumes was reported among older adults [86]. TNF-α has been shown to increase serotonin transporter activity in mice [87]. Patients with BD presented increased blood levels of inflammatory components, during acute affective episodes in particular [4,5,6,88,89,90], and levels of inflammatory constituents were found to be elevated in the postmortem brain [16,17] and CSF [13,14,15,91] of BD patients as compared to matched control subjects. A systematic review amalgamating 22 studies found a strong positive association between elevated IL-6 levels in blood, CSF and the postmortem brain and suicidal ideation and/or attempts or death [92]. Similarly, CSF IL-6 levels were found higher in those attempting violent versus nonviolent suicide, and positively correlated with future suicide completion [92]. Thus, using therapeutic interventions that reduce plasma and brain inflammatory mediator levels among BD patients may cause beneficial therapeutic effects. Therefore, we tested the effects of low-dose Li and low-dose aspirin on plasma and brain IL-6 and TNF-α levels of LPS-treated rats to explore their anti-inflammatory properties when administered each by themselves or in combination. We scrutinized these two cytokines because a large body of data links them to the pathophysiology and treatment of BD, as well as to other psychiatric conditions [4,5,13,14,15,17,88,89,91,92,93,94]. As summarized in Table 1, LPS administration resulted in a significant increase in plasma and brain IL-6 and TNF-α levels. The increase in IL-6 and TNF-α levels was partially reversed by Li and aspirin monotherapy, particularly in the HC. These findings are similar to the results of previous studies concluding that Li attenuates LPS-induced inflammation [19,46,48,77]. Khan et al. [77] found that Li significantly reduced the overexpression of TNF-α in the cortex and HC of LPS-treated rats. Beurel and Jope [95] reported that Li inhibited IL-6 production in plasma and brains of LPS-treated mice. Moreover, chronic treatment with low-dose aspirin also reversed some of the LPS-induced changes in cytokine levels (Table 1). Generally, aspirin alone exhibited a better anti-inflammatory profile than Li monotherapy (Table 1).

It is generally accepted that aspirin exerts anti-inflammatory effects through the inhibition of COX-2 and proinflammatory signaling pathways such as nuclear factor kappa (NF-κ)B when administered at relatively high doses, while its cardioprotective effects are obtained at lower doses via COX-1 inhibition [50]. Our results demonstrate that even at a low-dose, aspirin exhibits significant anti-inflammatory effects. Indeed, this finding is in line with the now recognized notion suggesting low-dose aspirin use in treating neuroinflammatory diseases [58,96,97]. A recent study found that treatment with low-dose aspirin reduces neuroinflammation in an animal model of multiple sclerosis [98]. Jung et al. [99] tested the effect of low-dose aspirin on activation of NF-κB in aged rats. Aspirin-treated rats presented lower NF-κB levels as compared to control rats, indicating suppressed NF-κB activity. Consistently, several studies suggested that COX-1 is imperative to microglial activation and consequent neuroinflammation [100,101], and that genetic deletion or pharmacological inhibition of COX-1 activity mitigates the inflammatory response [98,99,100,101,102,103,104,105,106]. Triflusal, a COX-1 inhibiting agent, decreased glial cell activation and proinflammatory cytokine production in a transgenic mouse model of Alzheimer’s disease [102]. Similarly, in rat models of cerebral ischemia, triflusal conferred a prominent neuroprotective effect through the inhibition of glial activation and suppression of NF-κB-regulated expression of IL-1β, TNF-α and COX-2 [103,104]. Choi et al. [105] reported that the selective COX-1 inhibitor SC-560 decreased glial stimulation and brain expression of inflammatory markers (e.g., TNF-α) in a mouse model of Alzheimer’s disease. Interestingly, the study [105] demonstrated that the selective inhibition of COX-1 suppressed GSK-3β activity through phosphorylation of its serine-9 residue (which downregulates the catalytic enzymatic activity), offering a possible mechanism by which COX-1 inhibition mitigates inflammation. In this regard, it is well recognized that inhibition of GSK-3β can mitigate the inflammatory response to LPS [107,108,109,110]. This is also pertinent to the pharmacological effects of Li, as numerous studies evinced that Li-induced GSK-3β inhibition contributes to the drug’s anti-inflammatory properties [22]. Furthermore, Dargahi et al. [111] reported that COX-1 inhibition decreased TNF-α and PGE2 levels in rat brain, reduced astrogliosis, and prevented neuronal cell death. Of note, COX-1 inhibition completely obliterated the induction of COX-2 in response to amyloid β-stimulated neuroinflammation, suggesting that COX-1 activity is required for COX-2 induction in response to neuroinflammatory stimuli [111]. Although chronic pretreatment with low-dose Li or aspirin each by themselves exerted limited effects on IL-6 and TNF-α plasma and brain levels in LPS-treated rats (Table 1), the combined treatment significantly and prominently diminished the cytokine levels both in plasma and the brain, implying enhanced protection against systemic inflammation. The mechanism underlying this augmented anti-inflammatory effect is yet to be unraveled. Previous studies have shown that Li, as well as other psychotropic drugs, inhibit the pro-inflammatory cellular pathway of NF-κB [18,22]. Similarly, several studies have demonstrated that aspirin attenuates NF-κB elicitation [99,112]. These findings corroborate the accumulating data attesting to altered NF-κB activity/levels in bipolar patients [18,89,113,114]. In preliminary rat experiments in our lab [Uzzan, Shvartsur and Azab–personal communication] exploring whether chronic low-dose Li or low-dose aspirin, as compared with their combination, influence NF-κB activity, FC, HT and HC nuclear p65 levels were measured. None of the treatment regimens reduced nuclear p65 levels, ruling-out an NF-κB inhibitory effect of the combination therapy.

Taking into account the reports of elevated IL-6 and TNF-α levels in BD patients, it is conceivable that therapeutic interventions that downregulate these cytokine plasma and brain levels in BD patients would, in turn, exhibit therapeutic benefits. Therefore, our findings that co-treatment with Li and aspirin lower IL-6 and TNF-α levels in LPS-treated rats, along with the encouraging behavioral effects of this treatment obtained in our previous study [60], prompted us to further investigate the behavioral therapeutic potential of the combination therapy. The significantly reduced immobility time and increased struggling duration induced by the combination therapy in the FST is suggestive of an antidepressant-like effect, corroborating the antidepressant-like and anti-anxiety-like effects of the combined treatment in our previous study [60]. Interestingly, in the present study, chronic Li monotherapy did not alter immobility (or struggling) time in the FST. This result is reminiscent to that of Bersudsky et al. [115]. In evaluating the immobility of Li treated mice, the variation of oral doses revealed that Li doses resulting in blood levels of 1.3 and 1.4 mEq/L induced a highly significant reduction in immobility time, while a dose rendering a blood level of 0.8 mEq/L (considered therapeutically-relevant in humans [80,81]) did not affect immobility time [115]. Unexpectedly, under the experimental conditions of the present study, no effect of Li (either by itself or together with aspirin) was obtained in the AHT, a broadly used animal model of an anti-manic potential of therapeutic interventions [68,69]. Nonetheless, it is worth noting that this model has several limitations, raising questions regarding its validity [116]. A possible explanation for the lack of an anti-manic/hyperactive-like effect of Li in the present study may derive from the trialed low-dose of Li. In clinical practice, higher plasma Li concentrations are required to ameliorate manic episodes than those required to treat bipolar depression [117]. Moreover, in line with our findings, a study of control healthy subjects revealed that Li did not reduce amphetamine-induced behavior [118]. Rodent studies reported conflicting effects of Li on amphetamine-induced hyperactivity [119,120,121,122,123]. Additional discrepancies of experimental conditions, e.g., sex and strain of the animals and/or characteristics of the experimental model may account for the inconsistent results in this regard [69,71,72,124,125,126,127,128,129,130].

## 5. Conclusions

The present study demonstrates that chronic co-treatment with low-dose Li and low-dose aspirin exerts enhanced anti-inflammatory effects accompanied by an antidepressant-like effect. Following the results of the present and previous study attesting to the safety and therapeutic potential of the combination therapy, it would be scientifically and clinically worthwhile to assess the tolerability and efficacy of this treatment regimen in randomized clinical trials among bipolar patients.

## Figures and Tables

**Table 1:** FC, HC, HT and plasma levels of inflammatory mediators IL-6 and TNF-α in Li- and/or aspirin-treated rats.

Group		Li vs. Control		ASA vs. Control		Li + ASA vs. Control		LPS vs. Control		LPS + Li vs. LPS		LPS + ASA vs. LPS		LPS + Li + ASA vs. LPS	
**Week**		**3**	**6**	**3**	**6**	**3**	**6**	**3**	**6**	**3**	**6**	**3**	**6**	**3**	**6**
**FC**	**IL-6**	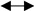	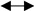	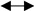	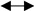	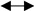	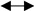	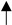	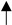	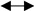	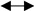	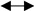	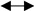	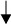	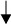
	**TNF-α**	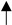	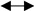	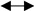	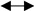	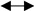	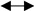	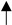	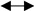	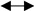	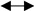	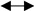	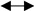	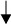	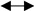
**HC**	**IL-6**	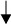	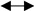	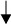	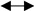	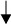	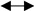	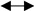	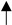	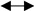	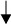	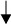	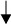	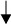	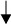
	**TNF-α**	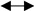	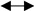	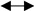	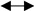	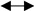	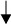	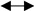	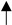	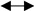	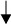	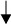	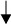	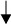	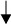
**HT**	**IL-6**	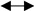	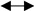	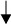	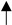	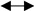	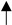	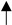	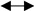	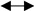	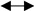	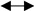	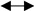	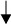	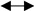
	**TNF-α**	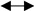	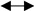	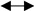	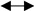	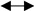	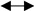	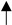	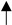	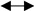	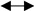	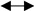	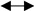	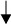	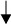
**Plasma**	**IL-6**	**UD**	**UD**	**UD**	**UD**	**UD**	**UD**	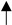	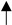	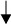	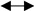	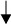	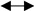	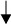	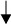
	**TNF-α**	**UD**	**UD**	**UD**	**UD**	**UD**	**UD**	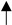	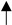	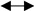	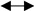	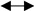	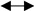	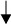	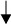

Comparisons were made between Li and/or aspirin treated rats across the different treatment conditions tested. The signs indicate the following trends: 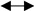
—a non-significant difference, 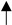
—a significant increase, 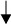
—a significant decrease. Abbreviations: ASA—acetylsalicylic acid, FC—frontal cortex, HC—hippocampus, HT—hypothalamus, IL—interleukin, Li—lithium, TNF—tumor necrosis factor.

## Data Availability

The datasets used and/or analyzed in the study are available from the corresponding author upon reasonable request.
